# The clinical assessment of amyotrophic lateral sclerosis patients’ prognosis by ZNF512B gene, neck flexor muscle power score and body mass index (BMI)

**DOI:** 10.1186/s12883-018-1219-9

**Published:** 2018-12-19

**Authors:** Chun-Jiang Yu, Li Wang, Sen-Lin Mao, Ying Zhang, Ling-Ling Song, Ling-Yu Cai, Ye Tao

**Affiliations:** 1 Department of Neurology, the Second Affiliated Hospital of the Harbin Medical University, Harbin, 150081 China; 2 Department of Geriatrics, the Second Affiliated Hospital of the Harbin Medical University, Harbin, 150081 China; 3 Department of Neurology, the Peoples’ Hospital of Hailar District, Hailar, 021000 China; 4 Department of Neurology, the Chao Yang Hospital, Liao Ning Provice, 122000 China; 5 Department of Neurology, the Peoples’ Liberation Army 117 Hospital of the Hangzhou, Zhe Jiang Province, 310000 China; 6 Department of Neurology, the Peoples’ Hospital of Sui Hua, Hei Longjiang Province, 152000 China

**Keywords:** Amyotrophic lateral sclerosis, Zinc finger protein 512B gene, Single nucleotide polymorphism, Risk allele, Neck flexor muscle power score, BMI, Prognosis

## Abstract

**Background:**

Assessment on the prognosis of amyotrophic lateral sclerosis (ALS) is becoming a focus of research in recent years since there is no effective treatment. The aim of the research is to explore the major factors involving in prognosis of ALS patients through long-term follow-up.

**Methods:**

ALS patients’ DNA extracted from peripheral blood white cells were detected for the risk allele by single nucleotide polymorphism (SNP) analysis. Neck flexor muscle score and body mass index (BMI) were recorded during Medical Research Council follow-up using manual muscle testing method.

**Results:**

ALS patients with risk alleles (C) deteriorated rapidly with poor clinical outcome. It seemed that the higher neck flexor muscle strength score in ALS patients with the longer survival time but without significant correlation (*p* > 0.05). The lower the basal body mass index, the shorter the survival time and the faster deterioration (*p* < 0.05). The patients with body mass index less than 22.04 seemed to have short survival time than those with BMI more than 22.04 (p < 0.05), however, the speed of deterioration in two groups of patients had no significant difference (*p* > 0.05).

**Conclusion:**

The risk (C) allele of the SNP (rs2275294) in the ZNF512B gene, cervical flexor muscle power and body weight index might have clinical potential for ALS prognostication, since these indicators is so simple to perform that they might be very suitable for primary clinics and even community medical institutions to carry out.

## Background

As a fatal progressive neurodegenerative disease, there is not any effective treatment for amyotrophic lateral sclerosis (ALS), the average survival duration might merely be 2 to 5 years. It brings heavy burden to family and society, and evenly a tough challenge to medical physicians who had been puzzled for decades. Since no advance could be expected in the predictable future years, the evaluation of its prognosis has become a more and more hot focus in research field in recent years. Recent studies showed that the risk (C) allele of the SNP (rs2275294) in the ZNF512B gene [1], neck flexor muscle power score [2] and body mass index (BMI) [3] are likely to become credible candidate evaluation indicator for ALS disease progression and clinical outcome. Although these findings had brought encouraging implication for medical staff [4], the most recent reports give out inconsistent or even paradoxical conclusion to some extent [5], it might be due to lack of comprehensive analysis and population sample basis. Whereas, there is few investigation involving Chinese population, especially concerning the above indicators’ combination. Therefore, the research aimed to explore the reliability indicators combination including risk (C) allele of the SNP (rs2275294) in the ZNF512B gene, neck flexor muscle power and body mass index, in order to establish reasonable pattern for ALS prognosis, and furthermore to imply appropriate medical intervention and health care as early as possible.

## Methods

### Subjects

This investigation was performed prospectively. This study was conducted in accordance with the declaration of Helsinki. This study was conducted with approval from the Ethics Committee of the Second Affiliated Hospital of the Harbin Medical University. Written informed consent was obtained from the participants. Enrollment criteria: All of the subjects was collected from Northeast Territory of China during 2012~2016 who were diagnosed as ALS according to both clinical symptoms, signs and electromyography (EMG) positive manifestation. The diagnosis was confirmed by two neurologist who had been engaged in clinical practice against ALS for years. All of the recruited patients fulfilled the diagnostic criteria of ALS China Neurology Branch of Chinese Medical Association [6]. Exclusion criteria: familial amyotrophic lateral sclerosis (fALS), frontotemporal dementia(FTD), severe cardiac, hepatic and renal function disorder, respiratory failure. Pulmonary function was evaluated every 3 months and noninvasive positive pressure ventilation (NIPPV) should be considered in case of insufficiency of respiratory such as forced vital capacity less than 80% of predicted value.

### Single nucleotide polymorphism analysis

To determine the risk allele, single nucleotide polymorphism analysis (SNP) for fragment (2275294) was performed. The specific primer (synthesis by the Shanghai Yingjun Biotechnology Co. Ltd.) was designed as follow: upstream: 5-TGCCAACTOTATTTGTCCATGT-3; downstream: 5-GCATGGAGGCTAGAGTGA-3. The genomic DNA of peripheral blood leukocytes was extracted and amplified by polymerase chain reaction (PCR). The reaction conditions of PCR were pre-denaturation at 94 °C for 5 min, denaturation at 94 °C for 10s,annealing at 61 °C for 10s, extension at 72 °C for 30s, 35 cycles totally, in the end and extension at 72 °C for 10 min finally. PCR amplification products were sequenced by direct sequencing of fluorescent dyes (Beijing infinite peaks), and SNP (rs275294) risk alleles were detected as CC, CT, TT genotypes.

### Cervical flexor muscle strength score

Cervical flexor muscle strength was determined using a handheld dynamometer. According to the MRC Research Council (Medical) scoring criteria, the cervical flexor muscle strength score is divided into 0–5 points. This study measured the neck flexor muscle is divided into 3 grades (5, 3–4 = 2).

### Body mass index (BMI)

Recorded height and weight at the time of diagnosis, and height and weight at follow-up, calculated body mass index, BMI = weight (kg)/height [2] (M2). According to the World Health Organization (WHO) standard, the body mass index is divided into 4 grades, low birth weight (BMI = 18.5), normal (BMI 18.6–249), overweight (BMI 25.0–299), obesity (BMI = 30); the changes of BMI were divided into three groups: BMl decreased by > 1 units, BMI changes the same, BMI increased > 1 units. Using the revised amyotrophic lateral sclerosis functional rating scale (ALSFRS-R) of ALS [7], diagnosis and follow-up were recorded. ALSFRS-R is composed of 12 parts, including language, writing, swallowing, sleep secretion, patients with fistula and cutting food tableware used function / gastrostomy in patients with fistula and the use of cutting food tableware, clothing and personal care, turning over in bed and finishing bedding, stair climbing ability, walking and breathing difficulties.

### Statistical analysis

Nonparametric test method and mean value ± standard deviationwere used to determine enumeration data and measurement data alternatively. Kaplan Meier survival curve with log rank test were employed to evaluate the impact of risk allele (C), cervical muscle strength score, body mass index on prognosis, *P* < 0.05 was considered as statistically significant, multivariate analysis was performed by the COX proportional hazards regression model, all the data was analysed by SPSS19.0 software.

## Results

### Base line data analysis

In this study 66 patients were followed up with 7 cases lost. Baseline characteristics were listed as below (Table 1).

**Table 1:** Baseline characteristics analysis

Characteristics	Live *N* = 25	Death *N* = 34	*P-*value	Statistic analysis method
Gender (male/female)	18/7	18/16	0.141	U-test
Age	52.08 ± 10.99	56.38 ± 8.30	0.092	T-test
Location of onset (bulb/limb)	8/17	11/23	0.977	U-test
Time from onset to diagnosis(months)	13.04 ± 10.67	15.50 ± 26.31	0.661	T-test
Riluzole treatment(yes/no)	18/7	15/19	0.035	U-test
Average ALSFRS-R score	44.32 ± 3.17	43.59 ± 2.58	0.333	T-test
Average survival time(months)	46.08 ± 24.20	49.03 ± 29.86	0.687	T-test
⊿FS	0.76 ± 1.60	0.65 ± 0.78	0.739	T-test
ventilator-assisted(yes/no)	17/8	21/13	0.212	U-test

### The prognosis of ALS patients with and without risk allele

**Fig. 1:**
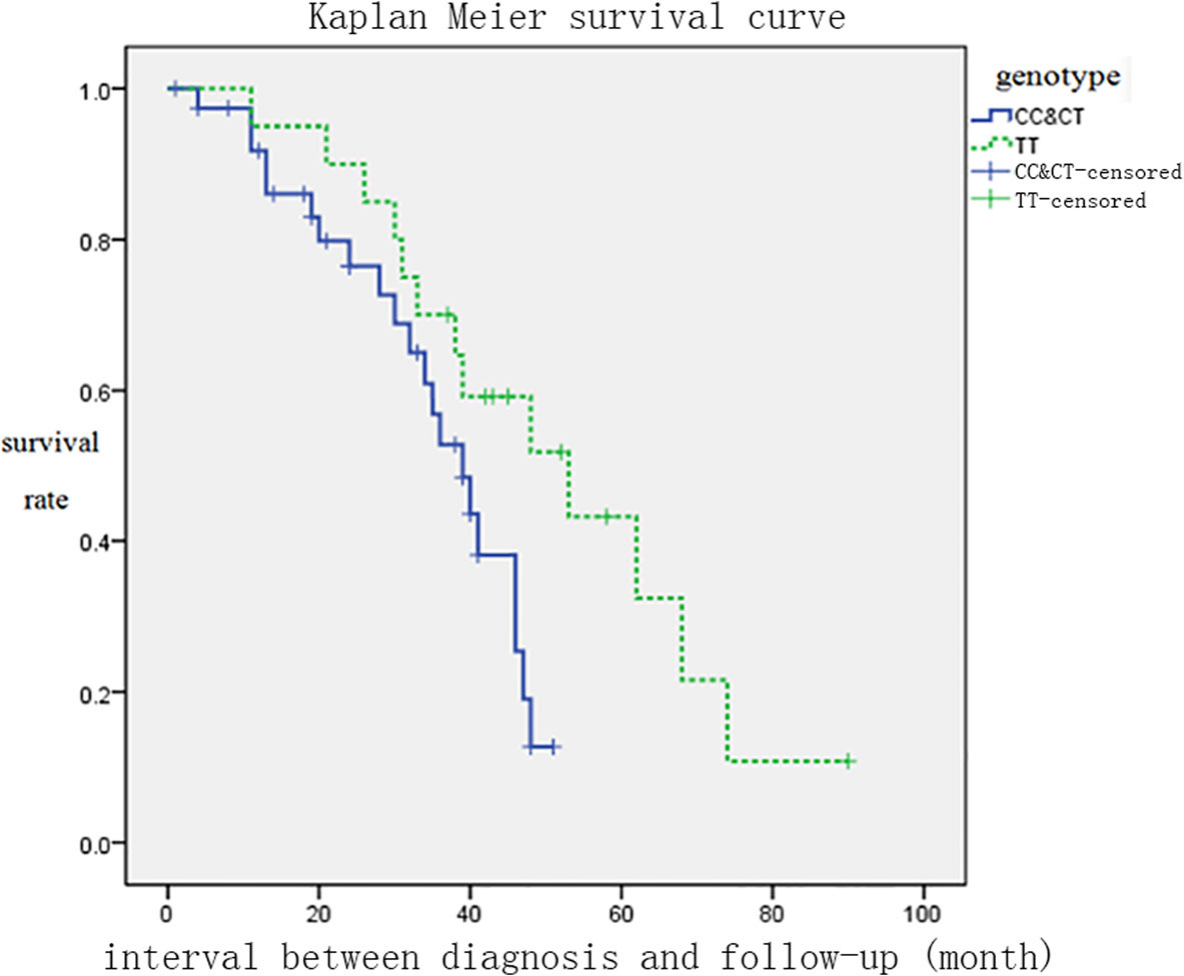
Kaplan-Meier survival curve of ALS patients with and without risk allele

**Fig. 2:**
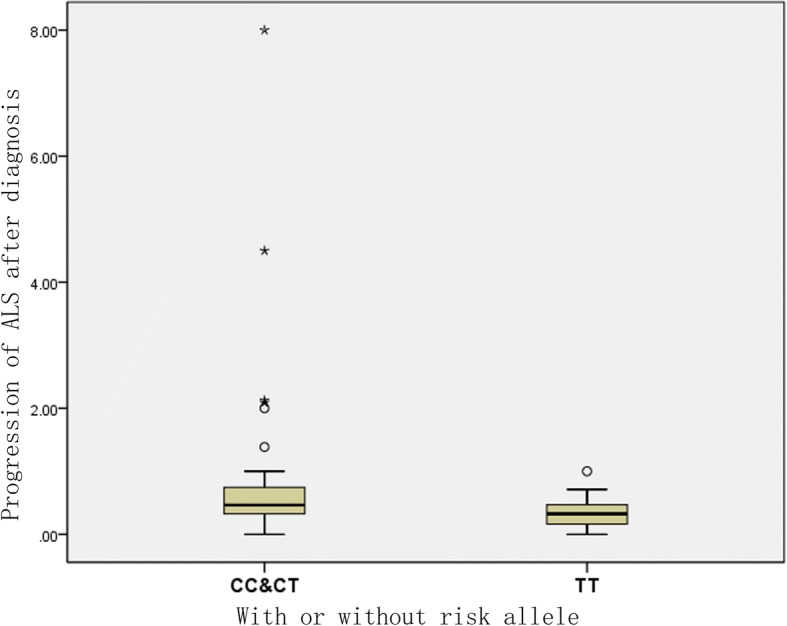
Rate of disease progression with and without risk allele

SNP analysis showed that there were three genotype in the ZNF512B gene SNP (rs2275294): CC, CT and TT. CC + CT is 39/59 (66.1%) and TT is 20/59 (33.9%). Among the 59 patients who were followed up, 30 died, 4 patients with ventilator occurred in 34 cases, end point events, including CC + CT 21/34 (61.76%), TT 13/34 (38.23%). The endpoint was considered when respiratory assistant was performed or death of subjects. Kaplan-Meier survival curve analysis showed that patients with risk alleles genotype (CC + CT) had shorter survival time and higher mortality rates than those without risk alleles and the difference was statistically significant (log-rank, *P* = 0.040, Fig. 1). Post-diagnosis disease progression (⊿FS) = (ALSFRS-R score at the time of diagnosis - follow-up ALSERS-R score)/diagnosis to follow-up time (month). The Mann-Whitney U test showed that patients with risk alleles genotype (CC + CT) had higher rates of disease progression than patients without risk alleles genotype (TT), with statistically significant differences (*P* = 0.022, Fig. 2).

### The cervical flexor muscle strength score

**Fig. 3:**
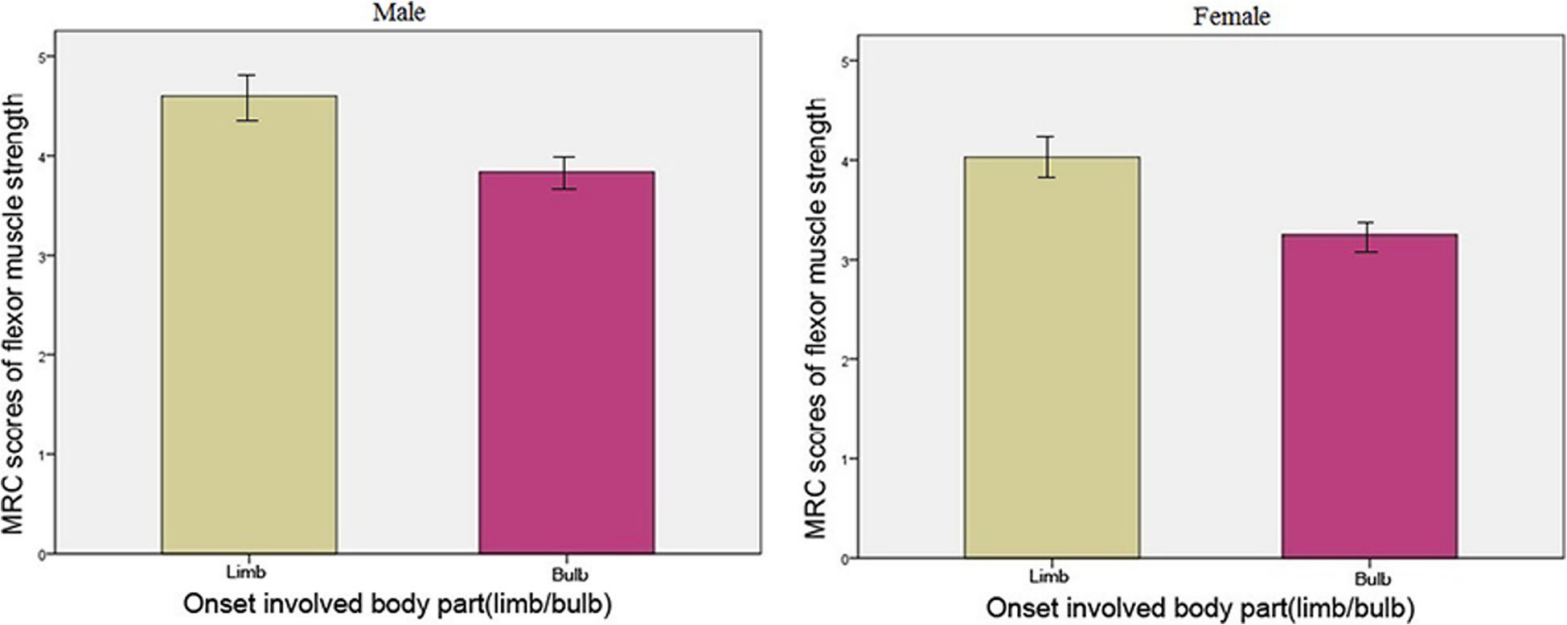
Neck flexor muscle score in bulb and limb involved ALS

According to the involved part of body, the cervical flexor muscle strength score in patients with bulb(medulla) involvement was markedly lower than those with limb(extremity) involvement (Kruskal-Wallis, *P* = 0.029) (Fig. 3).

**Fig. 4:**
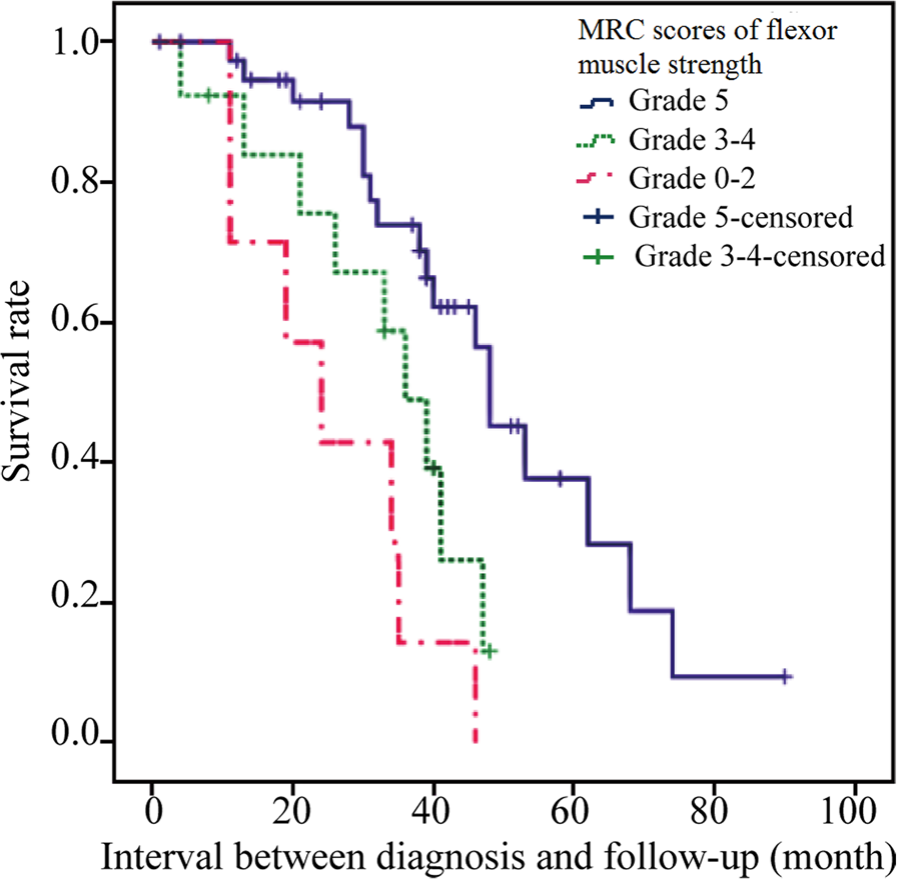
Kaplan-Meier survival curve according to neck flexor muscle score

Kaplan-Meier survival curve analysis shows the higher the neck flexor muscle strength score, the longer the survival time of ALS patients after diagnosis, the lower the mortality rate. The difference was satistically significant (log-rank test, *P* = 0.001 < 0.05) (Fig. 4).

There was no difference in the rate of disease progression between neck flexors of different grades. It does not yet show that the lower the neck flex or muscle strength score, the faster the disease progresses in ALS patient.

### Body mass index

**Fig. 5:**
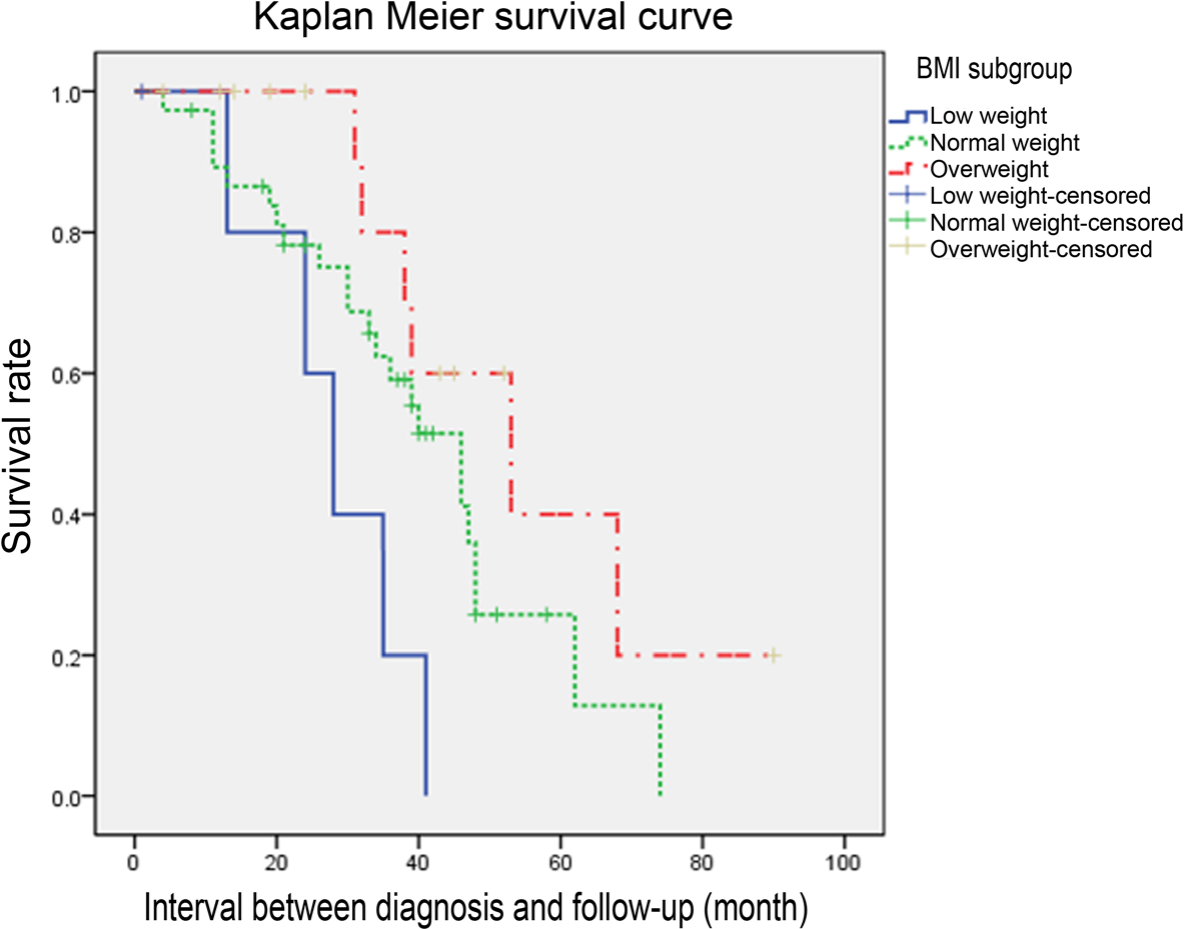
BMI analysis according to Kaplan-Meier survival curve

Kaplan-meier survival curve analysis showed that the lower the basal weight, the shorter the survival time and the higher mortality rate (*P* = 0.014 < 0.05). Pearson analyzed the relationship between basal body mass index and the rate of disease progression after diagnosis, but there was no significant correlation between them (r^2^ = 0.037) (Fig. 5).

**Fig. 6:**
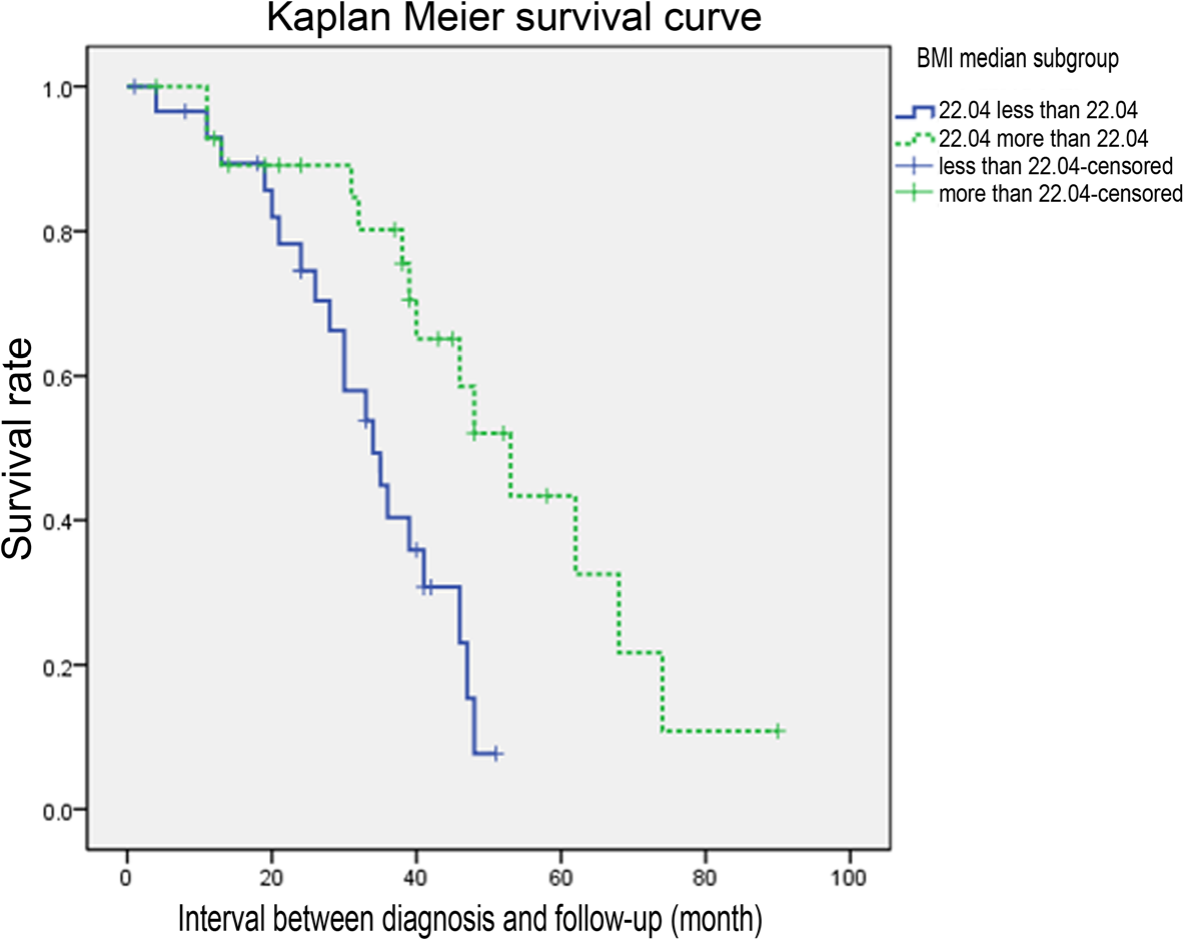
Analysis in Kaplan-Meier survival curve in subgroups divided by median value 22.04

The median follow-up of patients with weight index 22.04, grouped in a median of 22.04 Kaplan-Meier, survival curve analysis showed that BMI is less than 22.04 of the patients with BMI greater than 22.04 of the patients with short survival time, high mortality rate (log-rank, *P* = 0.03). There was no significant difference in the rate of disease progression between the two groups (T-test, *P* = 0.188) (Fig. 6).

The changes in body mass index were divided into three groups: the body mass index dropped by more than 1, the absolute value of body mass index was less than 1, and the BMI increased by more than 1. The Kaplan-Meier survival curve showed no significant difference in mortality between the three groups (log-rank, *P* = 0.087). Pearson analyzed the relationship between body mass index changes and the rate of disease progression, but there was no correlation between them (R^2^ = 0).

### COX risk ratio regression analysis

COX risk regression analysis of factors that might affect the prognosis of ALS showed that risk alleles (CC + CT) had a significant impact (*P* = 0.020) on risk alleles (CC + CT) and was an independent prognostic factor for ALS (HR 2.587; 95% CI 1.161~5.765).

## Discussion

### The risk (C) allele of the SNP (rs2275294) in the ZNF512B gene and prognosis of ALS

The exploration for candidate genes to predict ALS susceptibility and even its’ prognosis has been reasearch focus in recent years. Till now, more than 30 reports have proposed candidate genes as ALS susceptibility gene [8], but there is inconsistency to some extent or even controversy from findings of these research. In the project of genome-wide association studies (Genome Wide, Association Studies, GWAS), 5 susceptibility genes (FGGY, ITPR2, DPP6, KIFAP3 and UNC13A) and 2 loci (9p21.2 and 10q26.3) have been found in white people [9–12]. In 3 seperate studies [13, 14], the 9p21.2 locus was repeatedly noted, however, this locus was not detected in ALS patients from China and Japan [15]. Chio and colledges reported protective effect of UNC13A gene on ALS in Italy population by single nucleotide polymorphism analysis [16], but the mechanism of the protective effect is still uncertain.

In Asian population, Iida et al. [15] had performed SNP analysis using Gene Bank of Japanese ALS patients. A functional fragment SNP (rs2275294) which is located between rs2252258 and 20q13.33 of rs816953 with a length of 111 kb was found to be significantly associated with ALS prognosis by gene mapping. Comparing with the findings from the Japanese people, the study focus on Chinese population is attracting more attention because of its much more sample. However, interestingly the results from different centers gives out inconsistent and even contradict conclusions [5] which imply this research field is worthy of further exploration.

According to the results of our study showing ALS patients with SNP (rs2275294) containing risk allele (C) lives shorter survival time, high mortality rate and more rapid deterioration. The findings are similar to the previous reports from 176 Japanese patients with ALS. It can be dedicated that the SNP (rs2275294) containing risk allele (C) is a reliable predictor for the prognosis of ALS at least in east Asian. What’s more, the findings should be further verified in populations from other parts of the world.

### The cervical flexor muscle strength

Our findings showed that the higher the cervical flexor muscle strength score, the longer the survival time of patients with ALS. The lower neck flexor muscle strength was more often seen in the patients with the onset of medulla oblongata resulting in dysphagia,dysphonia and tongue muscle atrophy rather than those with limb onset.

The relationship between carotid flexor muscle score and prognosis in patients with ALS was firstly proposed by Nakamura [2], in which prospective multicenter cohort study were performed on 401 patients, implying that neck flexor muscle strength score is an independent and important prognostic predicting factor in ALS comparing with other common risk factors. In terms of survival time, our results are consistent with Nakamura’s findings. Interestingly, our findings showed that the bulbar onset patients seem to have lower neck flexor muscle strength score. The mechanism might be due to adjancent of neck flexors and respiratory muscles in medullar which are part of the common innervation resulting in joint innervation and spacing spread of degeneration of neurons.

The amplitude of phrenic nerve action potential was reported as an important prognostic indicator of ALS [17], suggesting that the weakness of the flexor tendon in the neck is related to diaphragmatic weakness which contribute to respiratory failure to some extent [18]. However, due to its limitations to clinical practice, the predict value of this indicator should be evaluated further.

### Body mass index

Since BMI can be determined so simply to be a useful indicator of ALS prognosis, the relationship between body mass index and ALS prognosis has been a hot point in research field. Previous studies found that people with normal BMI and low BMI were 2.48 times more likely to develop ALS than obese ones, and that the risk of ALS increased by 6% with BMI per unit dropped [19–21]. Abel and Chio also drawn similar conclusion, and pointed out that ALS had a higher incidence in professional athletes, and the decrease of BMI might result in the shortened survival time of ALS patients [22, 23]. In addition, Jawaid^3^ proposed that the foundation of BMI is the risk factor of ALS with age, disease progression and survival time and the BMI change is the factor predicting the progression and survival of ALS. Our findings showed that the basal BMI should be viewed as an independent ALS prognostic predicting factor which is consistent with previous reports, suggesting that stable BMI without or with slow loss of weight might prolong life expancy of ALS. The underling mechanism was recently presumed due to the metabolic states in which impaired insulin signaling could be involved suggesting the role of insulin resistance in the pathogenesis of ALS [24].

## Conclusion

To sum up, owing to their easy accessibility, the risk allele, cervical flexor muscle strength and BMI should be expected to be appropriate ALS prognostic indicators to provide reliable advice to physician and health care in clinical practice in future. However, since the sample of this research is relatively small, further investigation should be carried out in large scale of population.

## Abbreviations

ALS: Amyotrophic lateral sclerosis
BMI: Body mass index
EMG: Electromyography
fALS: familial amyotrophic lateral sclerosis
FTD: Frontotemporal dementia
NIPPV: Noninvasive positive pressure ventilation
PCR: Polymerase chain reaction
SNP: Single nucleotide polymorphism
SNP: Single nucleotide polymorphism analysis
WHO: World Health Organization
